# Evaluation of Computed Tomography Scanners for Feasibility of Using Averaged Hounsfield Unit–to–Stopping Power Ratio Calibration Curve

**DOI:** 10.14338/IJPT-17-0035.1

**Published:** 2018-11-30

**Authors:** Heeteak Chung, Sina Mossahebi, Arun Gopal, Giovanni Lasio, Huijun Xu, Jerimy Polf

**Affiliations:** Department of Radiation Oncology, University of Maryland, Baltimore, MD, USA

**Keywords:** stoichiometric calibration, animal tissue surrogate, water-equivalent thickness

## Abstract

**Purpose:**

The purpose of this study was to quantify the variability of stoichiometric calibration curves for different computed tomography (CT) scanners and determine whether an averaged Hounsfield unit (HU)–to–stopping power ratio (SPR) calibration curve can be used across multiple CT scanners.

**Materials and Methods:**

Five CT scanners were used to scan an electron density phantom to establish HU values of known material plugs. A stoichiometric calibration curve was calculated for CT scanners and for the average curve. Animal tissue surrogates were used to compare the water-equivalent thickness (WET) of the animal tissue surrogates calculated by the treatment planning system (TPS) and the WET values measured with a multilayered ionization chamber. The calibration curves were optimized to reduce the percentage of difference between measured and TPS-calculated WET values. A second set of tissue surrogates was then used to evaluate the overall range of uncertainty for the optimized CT-specific and average calibration curves.

**Results:**

Overall, the average variation in HU for all 6 calibration curves before optimization was 8.3 HU. For both the averaged and CT-specific calibrations, the root mean square error (RMSE) of the percentage of difference between TPS-calculated and measured WET values before optimization was 4%. The RMSE of the percentage of difference for the TPS-calculated and multilayered ionization chamber measured WET values after the optimization for both averaged and CT-specific calibration curves was reduced to less than 1.5%. The overall RMSE of the TPS and the measured WET percentage of difference after optimization was 2.1% for both averaged and CT-specific calibration curves.

**Conclusion:**

Averaged CT calibration curves can be used to map the HU-to-SPR in TPSs, if the variations in HU values across all scanners is relatively small. Performing tissue surrogate optimization of the HU-to-SPR calibration curve has been shown to reduce the overall uncertainty of the calibration for averaged and CT-specific calibration curves and is recommended, especially if an averaged HU-to-SPR calibration curve is used.

## Introduction

Calibration of computed tomography (CT) scanners for use in proton radiotherapy treatment planning is most commonly accomplished with the stoichiometric method proposed by Schneider et al [1]. That method relates the CT scanner's Hounsfield unit (HU) values for a given tissue in a CT image to the ratio of the stopping power of the tissue relative to water or, simply, the stopping-power ratio (SPR) of the tissue. The stoichiometric calibration method is designed to create an HU-to-SPR calibration curve in the treatment planning system (TPS) for a given CT scanner to estimate the water-equivalent thickness (WET) and to determine the proton range [2].

The stoichiometric calibration method has been widely adopted for clinical calibration; however, several factors contribute to uncertainty in the determination of SPR values. The main sources of uncertainty are deviation of HU values because of CT image artifacts and beam-hardening effects, variation of the linear regression fit in the stoichiometric formalism, deviation of the human body tissue from International Commission on Radiation Units and Measurements (ICRU) reference tissues, and variation in the mean excitation energies needed to calculate the SPRs [2]. Additionally, the HU-to-SPR calibration curves are very sensitive to changes in the energy spectrum of the x-ray beam of the CT scanner [2–4]. Deviations in the HU values for energy spectra produced from a CT scanner for the same material can be up to 3% [2], and the variation between CT scanners for the same material can be up to 10% [5]. Therefore, a small difference in the HU, especially in the lung and bone regions of the calibration curve, can produce significant changes in the SPR value, which can, in turn, have clinically significant effects on the dose and range of the proton beam delivered to the patient [6, 7].

Because of the differences in the beam characteristics (x-ray energy spectra, detector response, and the like) among CT scanners, it is often recommended that a separate HU-to-SPR calibration be performed for each CT scanner. The need for multiple HU-to-SPR calibration curves for multiple CT scanners can lead to many calibration curves available for dose calculation within the TPS. For example, within the Department of Radiation Oncology at the University of Maryland (Baltimore), there are 5 clinically operational CT scanners. Four scanners are in the photon clinics (at the university hospital and in 3 community practice clinics) and 1 is at the proton clinic (Maryland Proton Treatment Center, Baltimore). Our proton facility uses a ProBeam proton therapy system (version 3.0, Varian Medical Systems, Palo Alto, California) that accelerates protons with a cyclotron and delivers proton beams with an energy range of 70 to 245 MeV inside the treatment rooms. The system provides pencil-beam scanning with magnets and a narrow proton beam to deliver discrete spots of protons in a 2-dimensional plane to paint a tumor layer by layer. Because of the structure of the department, we find that some of our patients may receive planning CT scans from a photon clinic and, subsequently, have their planning and treatment at the proton facility. This was the main motivation for undertaking the current study.

The purpose of this study was to quantify the variability of the stoichiometric calibration curves for different CT scanners and to determine whether an averaged HU-to-SPR calibration curve could be used across multiple scanners. The uncertainty in the HU-to-SPR calibration curves for each CT scanner and for the averaged HU-to-SPR curve were evaluated with animal-tissue surrogates. We applied an optimization process to each curve thus obtained to minimize uncertainties in the calibration and generated a final averaged HU-to-SPR curve. The overall uncertainty in applying the optimized averaged HU-to-SPR calibration curve for dose calculation on images from each individual CT calibration curve was then evaluated on a separate set of animal tissue surrogates.

## Materials and Methods

### CT Scanners and CT Calibration Phantom

There are 5 CT scanners in service at the Department of Radiation Oncology at the University of Maryland. There are 4 Brilliance Big Bore scanner (Philips Medical Systems, Best, the Netherlands) and 1 Definition Edge scanner (Siemens, Erlangen, Germany). All the Philips CT scanners are located in the photon clinic, and the Siemens CT scanner is located in the proton clinic. To establish the HU values with known materials, an electron density phantom (model 062M, CIRS, Carlton, Victoria, Australia) containing 7 tissue-equivalent electron density plugs and 1 water vial [8] in an abdominal configuration, with 2 nested disks combined, was imaged with all 5 CT scanners. Elemental composition and the density of the plugs were obtained from the phantom manufacturer. All 5 CT scanners used similar scanning protocols; 120 peak kilovoltage, 3-mm slice thickness, and 500-mm field of view. All the images were transferred to the Varian Eclipse TPS (version 13.7) to obtain the measured HU values from the plugs. We obtained 6 sets of HU values for the electron density phantom: 5 from the CT scanners, and 1 from the averaged HU values from the 5 CT scanners.

### Stoichiometric Calibration

To generate the HU-to-SPR calibration curves for the 5 CT scanners and averaged HU values, the stoichiometric calibration method proposed by Ainsley and Yeager [3] was used. To summarize briefly, the electron density phantom and the plugs were scanned and the measured HU values for the plugs were obtained. The measured HU values for each plug were used to determine the parameterization coefficients A, B, C, and D via linear regression fit:

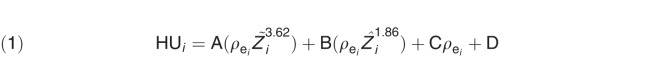



The coefficient D and 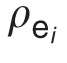 are the intercept and the relative electron density to water for the *i*th plug, respectively. The coefficients A, B, and C of each plugs represent the parameterized contributions of the linear attenuation coefficient from the photoelectric effect, coherent scattering, and Compton scattering, respectively. *Z̃_i_* and *Ẑ_i_* are the effective atomic numbers, which are defined as follows:

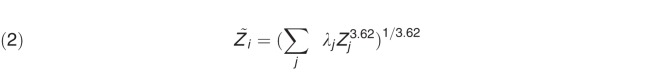


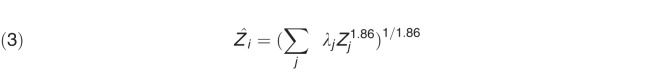
where 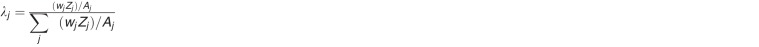. The terms *w_j_*, *Z_j_*, and *A_j_* are the mass fraction, the atomic number, and the mass of element *j* in plug *i*. By taking the parameterized coefficients and reference tissue compositions from ICRU reports [9, 10] into consideration, theoretical HU values were computed for each reference tissue. The stopping power ratio, SPR_calc_, for each reference tissue was then calculated using the Bethe-Bloch equation [11]:

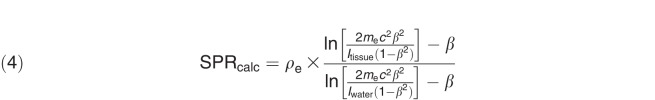
where *m*_e_*c*^2^ is the rest mass energy of the electrons, β is the relativistic speed of the proton with respect to the speed of light *c*, and *I*_tissue_ and *I*_water_ are the mean ionization energy of the reference tissue and water, respectively. The ionization energy for each element was adopted from Seltzer and Berger [12], and the mean ionization energy of a tissue composition was calculated using the Bragg additivity rule [1]. A fixed proton energy of 240 MeV was used to compute the relativistic speed of the proton in equation 4, assuming the SPR is approximately constant with the proton energy in the therapeutic region [2, 10]. Finally, the theoretical HU for each reference tissue was plotted against its calculated SPR to generate the HU-to-SPR calibration curve. Of all the reference tissues, selected tissues were connected piecewise in the general regions that represent lung, tissue, water, and bone to create a calibration curve. The curve was extrapolated to HU = −1000 to include air. This process was repeated for each of the 5 CT scanners, and the average of the HU values computed for each CT scanner was calculated and used to plot the average HU-to-SPR curve.

### Evaluation of HU-to-SPR Calibration Curves

After the initial HU-to-SPR calibration, a set of animal tissue samples, referred to as *preoptimized tissue surrogates*, was used to evaluate and optimize all 6 HU-to-SPR calibration curves (5 CT scanner-specific curves and 1 averaged calibration curve). For the optimization of the calibration curves, the preoptimized tissue surrogates were the water, fat (adipose tissue), muscle, intact stomach, liver, femur, and head of a pig. Except for water, all other tissue surrogates were placed in a hermetically sealed bag and frozen. All the tissues were scanned on the a Siemens Definition Edge CT scanner only because of the relative ease of access to the scanner. The same CT protocols used to scan with the CIRS phantom were used to acquire images of the tissue surrogates. Once scanned, the images were imported to the Varian Eclipse TPS, and all the HU-to-SPR calibration curves were applied to the images to obtain the WET value for each tissue. Next, the WET value for each tissue surrogate was measured with a multilayered ionization chamber (MLIC; Giraffe, IBA Dosimetry GmbH, Schwarzenbruck, Germany) with a proton-pencil beam energy of 240 MeV. The WET values were obtained by taking the difference between the distal 80% of the Bragg peak measured with the MLIC with and without the tissue:

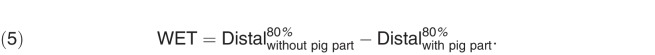



The uncertainty in the SPR-to-HU calibration curves was then defined as the percentage of the difference in the TPS-based and measured WET values: 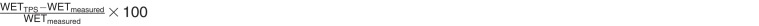


The measurement uncertainty of the MLIC was 0.4 mm, based on internal experience from current and other studies. To minimize setup errors for the MLIC measurements, BBs were placed on the tissue surrogates to help with the setup.

Based on the results of the SPR-to-HU uncertainty, all 6 calibration curves were optimized to minimize the percentage of difference between the TPS-based and measured WET values. To minimize the percentage of difference between the TPS-based and measured WET values, the SPR-to-HU calibration curves were adjusted overall for optimization. When performing adjustments, only the SPR values were adjusted, whereas the HU values remained static. To better fit the curves with the measured results, 2 arbitrary points were added (indicated as *Fill in* in **Table 3**) to the curves.

### Evaluation of the Overall Uncertainty of the Optimized Calibration Curves

After the tissue surrogate–based HU-to-SPR calibration curve optimization was performed for all 6 calibration curves, a separate tissue surrogate sample, which we call the *postoptimized tissue surrogate*, was used to evaluate the overall uncertainty of the calibration curves. The postoptimized tissue surrogate included muscles, ribs, fat (adipose tissue), and cartilage from a pig, intact leg (including muscle cartilage and bone) from a chicken, and air cavities. All the tissues were placed in a Tupperware container (Tupperware Brands, Orlando, Florida) and frozen for CT scanning. The arrangement of the surrogate tissues in the container was performed randomly, on purpose, with air cavities to simulate the true human anatomy as closely as possible. The postoptimized tissue surrogate sample was imaged with all 5 CT scanners with the protocols described in the previous section. Once imaged, the tissue surrogate was irradiated with a 240 MeV proton beam at 6 different locations within the container, using the MLIC to determine 6 WET measurement points for each CT image. The proton beam energy of 240 MeV was chosen to ensure that there was enough energy to penetrate through the tissue surrogates and maintain a stable and high dose rate. The locations of the 6 measurement points are identified in **Table 4** (xy-plane is the axial plane, xz-plane is the coronal plane, and yz-plane is the sagittal plane). The measurements along the thin side of the tissue sample is the x-direction (5 measurements) and the thicker side of the tissue sample is the z-direction (1 measurement). The relative position of the axial measurements are indicated in **Table 5**.

To evaluate the overall uncertainty of the averaged CT calibration curve, the optimized, averaged HU-to-SPR calibration curve was applied to the images from the 5 CT scanners. The WET values predicted by the TPS for each tissue surrogate were obtained and evaluated against the measured WET values. For the CT-specific calibration evaluation, the CT-specific, optimized HU-to-SPR calibration curves were used for the given CT scan to obtain the WET values from the TPS for each scanner.

## Results

### Evaluation of Preoptimized Tissue Surrogate–Adjusted Calibration Curves

**Figure 1.**
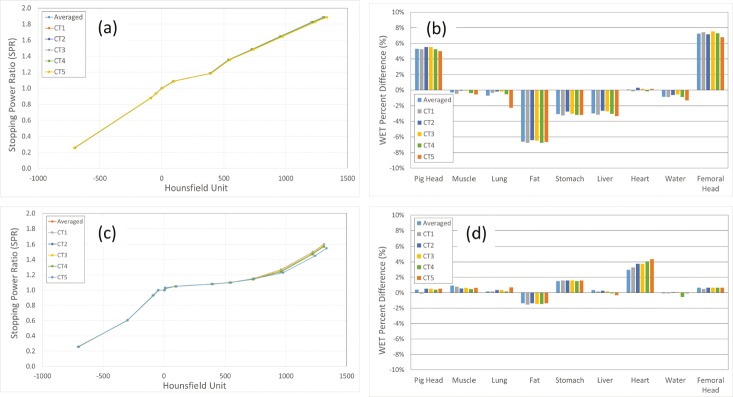
(a) Preoptimized tissue surrogate–adjusted calibration curve. (b) Initial calibration (preoptimized tissue surrogate). (c) Postoptimized tissue surrogate–adjusted calibration curves. The 2 fill-in points are shown with red arrows. (d) Postoptimized tissue surrogate. Water-equivalent thickness (WET) percentage of difference [WET% = (WET_TPS_ − WET_measurement_)/(WET_measurement_)] of WET values for the preoptimized tissue surrogates between treatment planning system and the measurements for 6 calibration curves.

For the evaluation of the 6 calibration curves, 10 ICRU reference tissues, representing lung, soft-tissue, and bone, were chosen. The selected reference tissues were lung-inflated, adipose tissue (ICRU 49), water, cartilage, humerus, sacrum (female), rib-10th, mandible, cortical bone (ICRU 49) and cortical bone. A plot of all 6 preoptimized tissue surrogate–adjusted calibration curves is shown in **Figure 1a**. The overall average variation (2σ) of the HU values for the 6 pretissue surrogate–adjusted calibration curves was 8.3 HU with maximum and minimum HU values of 22.2 HU and 2.2 HU, respectively (see **Table 1**).

**Table 1:** Selected reference tissues from the International Commission on Radiation Units and Measurements (ICRU) report 46 [9] with corresponding stopping power ratio (SPR; before optimization) and Hounsfield unit (HU) values. Maximum (Max), minimum (Min), average, and average variation (2σ) of the 6 HU values from the calibration curves for each reference tissue are tabulated.

**Tissue**	**SPR**	**CT1**	**CT2**	**CT3**	**CT4**	**CT5**	**Average**	**Max**	**Min**	**2σ (HU)**
Lung inflated	0.257	−701.0	−703.0	−699.9	−701.0	−706.5	−702.3	−699.9	−706.5	4.7
Adipose tissue (ICRU report 49 [10])	0.878	−86.4	−89.1	−85.9	−86.4	−90.8	−87.7	−85.9	−90.8	3.8
Breast, 33-67	0.904	−67.4	−70.2	−67.7	−67.4	−70.5	−68.6	−67.4	−70.5	2.8
Breast, 50-50	0.933	−44.9	−47.9	−46.0	−44.9	−46.5	−46.1	−44.9	−47.9	2.2
Water	1.000	3.3	0.1	−0.2	3.3	6.3	2.6	6.3	−0.2	4.8
GI tract	1.019	26.4	23.2	23.3	26.4	28.7	25.6	28.7	23.2	4.2
Bladder, empty	1.030	39.0	35.8	35.7	39.0	41.8	38.3	41.8	35.7	4.6
Kidney	1.036	45.2	42.0	42.0	45.2	47.7	44.4	47.7	42.0	4.4
Blood	1.047	56.4	53.2	53.1	56.4	59.1	55.6	59.1	53.1	4.5
Skin	1.068	75.8	72.9	74.5	75.8	77.7	75.3	77.7	72.9	3.2
Cartilage	1.087	97.3	94.0	93.0	97.3	101.8	96.7	101.8	93.0	6.2
Humerus	1.186	391.3	388.7	386.1	391.3	397.2	390.9	397.2	386.1	7.4
Femur	1.291	479.9	477.1	474.8	479.9	485.6	479.5	485.6	474.8	7.3
Sacrum (female)	1.355	540.6	537.7	534.4	540.6	548.4	540.4	548.4	534.4	9.3
Rib, second and sixth	1.375	570.8	567.9	564.3	570.8	579.1	570.6	579.1	564.3	9.8
Vertebrae, C4	1.385	584.3	581.3	577.7	584.3	592.8	584.1	592.8	577.7	10.0
Rib, 10th	1.485	730.7	727.7	722.7	730.7	741.6	730.7	741.6	722.7	12.4
Cranium	1.576	863.2	860.3	854.0	863.2	876.4	863.4	876.4	854.0	14.6
Mandible	1.645	961.3	958.4	951.2	961.3	976.2	961.7	976.2	951.2	16.3
Cortical bone (ICRU report 49 [10])	1.825	1219.6	1216.7	1207.2	1219.6	1238.6	1220.3	1238.6	1207.2	20.4
Cortical Bone	1.885	1313.0	1310.1	1299.7	1313.0	1333.8	1313.9	1333.8	1299.7	22.2

**Abbreviation:** 33-67, 33% fibroglandular tissue–67% fat; 50-50, 50% fibroglandular tissue–50% fat; CT, computed tomography.

A comparison of the TPS-calculated WET to the measured WET from the tissue surrogates is shown in **Figure 1b**, with the tabulated, preoptimized values shown in **Table 2**. The tissues that showed the largest WET percentage of difference were femoral head (range, 6.8% to 7.6%), fat (range, −6.4% to −6.8%), and intact pig head (range, 5.0% to 5.5%). The positive sign on the WET percentage of difference shows that the measured WET value was smaller than TPS WET value. A negative sign shows that the measured WET value was larger than TPS WET value. The root mean square errors (RMSEs) of the WET uncertainty for the preoptimized tissue surrogates for the averaged and the CT-specific calibration curves were both 4%. The overall RMSEs for both the averaged and CT-specific calibration curves were also 4%. It was determined that the overall SPR uncertainty without the animal tissue surrogate adjustment was approximately 4% for this study.

**Table 2:** Preoptimized and postoptimized tissue surrogate–adjusted water-equivalent thickness (WET) percentage of differences between treatment planning system (TPS) and measured WET values.

**Tissue**	**Preoptimized tissue surrogate adjustment (%)**						**Postoptimized tissue surrogate adjustment (%)**					
	**Average**	**CT1**	**CT2**	**CT3**	**CT4**	**CT5**	**Average**	**CT1**	**CT2**	**CT3**	**CT4**	**CT5**
Pig head	5.3	5.0	5.3	5.5	5.5	5.3	0.4	0.5	−0.1	0.5	0.5	0.4
Muscle	−0.3	−0.5	−0.5	−0.1	−0.1	−0.4	0.9	0.6	0.8	0.6	0.5	0.5
Lung	−0.7	−2.3	−0.3	−0.2	−0.2	−0.5	0.2	0.7	0.2	0.3	0.3	0.2
Fat	−6.6	−6.7	−6.8	−6.5	−6.4	−6.8	−1.4	−1.4	−1.5	−1.5	−1.4	−1.5
Stomach	−3.1	−3.2	−3.3	−3.0	−2.7	−3.2	1.5	1.6	1.6	1.6	1.6	1.5
Liver	−3.0	−3.3	−3.1	−2.7	−2.6	−3.1	0.3	−0.3	0.2	0.2	0.3	−0.1
Heart	0.1	0.2	−0.2	0.2	0.3	−0.2	3.0	4.4	3.3	3.7	3.7	4.1
Water	−0.9	−1.3	−0.9	−0.6	−0.6	−0.9	−0.1	−0.1	−0.1	0.1	0.1	−0.6
Femoral head	7.2	6.8	7.4	7.6	7.2	7.3	0.6	0.6	0.5	0.6	0.6	0.6

**Abbreviation:** CT, computed tomography.

**Table 3.** Postoptimized tissue surrogate–adjusted calibration curves.

**Tissue**	**Averaged**	**SPR**	**CT1**	**SPR**	**CT2**	**SPR**	**CT3**	**SPR**	**CT4**	**SPR**	**CT5**	**SPR**	**Min (HU)**	**Max (HU)**	**2σ (HU)**
Lung, inflated	−702.3	0.257	−701.0	0.257	−703.0	0.257	−699.9	0.257	−701.0	0.257	−706.5	0.257	−699.9	−706.5	2.4
Fill in	−300.0	0.605	−300.0	0.605	−300.0	0.605	−300.0	0.605	−300.0	0.605	−300.0	0.602	−300.0	−300.0	0.0
Adipose tissue (ICRU report 49 [10])	−87.7	0.930	−86.4	0.930	−89.1	0.930	−85.9	0.930	−86.4	0.930	−90.8	0.930	−85.9	−90.8	1.9
Breast, 50-50	−46.1	1.000	−44.9	1.000	−47.9	1.000	−46.0	1.000	−44.9	1.000	−46.5	1.000	−44.9	−47.9	1.1
Water	2.6	1.000	3.3	1.000	0.1	1.000	−0.2	1.000	3.3	1.000	6.3	1.000	6.3	−0.2	2.4
Fill in	10.0	1.030	10.0	1.030	10.0	1.020	10.0	1.020	10.0	1.020	10.0	1.030	10.0	10.0	0.0
Cartilage	96.7	1.050	97.3	1.050	94.0	1.050	93.0	1.050	97.3	1.050	101.8	1.050	101.8	93.0	3.1
Humerus	390.9	1.080	391.3	1.080	388.7	1.080	386.1	1.080	391.3	1.080	397.2	1.080	397.2	386.1	3.7
Sacrum (female)	540.4	1.100	540.6	1.100	537.7	1.100	534.4	1.100	540.6	1.100	548.4	1.100	548.4	534.4	4.6
Rib, 10th	730.7	1.150	730.7	1.150	727.7	1.142	722.7	1.146	730.7	1.142	741.6	1.150	741.6	722.7	6.2
Mandible	961.7	1.270	961.3	1.270	958.4	1.242	951.2	1.250	961.3	1.242	976.2	1.230	976.2	951.2	8.1
Cortical bone (ICRU report 49 [10])	1220.3	1.500	1219.6	1.500	1216.7	1.472	1207.2	1.470	1219.6	1.472	1238.6	1.450	1238.6	1207.2	10.2
Cortical bone (ICRU report 49 [10])	1313.9	1.600	1313.0	1.600	1310.1	1.572	1299.7	1.570	1313.0	1.572	1333.8	1.550	1333.8	1299.7	11.1

**Abbreviations:** 2σ, average variation; 50-50, 50% fibroglandular tissue–50% fat; CT, computed tomography; HU, Hounsfield unit; ICRU, International Commission on Radiation Units and Measurements; Max, maximum; Min, minimum; SPR, stopping power ratio.

**Table 4.** The overall uncertainty of the calibration curves. There were 6 points of measurements, which are shown in the “Relative location” column. For the averaged computed tomography (CT) calibration evaluation, the averaged CT calibration curve was applied for all 5 CT images to obtain the water-equivalent thickness (WET) values from the treatment planning system (TPS). For the CT-specific calibration evaluation, the CT-specific calibration curves were applied to a given CT scan to obtain the WET values from the TPS.

**Relative location (cm)**	**Averaged CT calibration (%)**					**CT-specific calibration (%)**				
	**CT1**	**CT2**	**CT3**	**CT4**	**CT5**	**CT1**	**CT2**	**CT3**	**CT4**	**CT5**
*X* = 0	−2.8	−0.6	−2.3	−0.1	−1.2	−3.0	−0.5	−2.5	−1.3	−1.6
*X* = −4.1	−3.7	−3.0	−3.7	−3.3	−3.5	−4.3	−4.0	−3.3	−3.4	−3.7
*X* = −8.4	−2.2	−0.7	−0.8	1.2	0.1	−2.2	−0.8	−1.7	−0.1	−0.2
*X* = −13.4	−2.1	1.2	0.5	1.6	1.2	−1.5	0.9	0.6	1.8	0.9
*X* = −17.9	−0.2	0.1	−0.4	0.3	−0.6	−1.9	−0.7	−0.6	−0.7	−0.8
*Z* = 2	2.4	2.6	2.6	3.4	2.8	1.9	2.0	2.3	2.4	2.3
RMSE	2.5	1.7	2.1	2.1	2.0	2.7	1.9	2.1	2.0	2.0

**Abbreviation:** RMSE, root mean square error.

### Evaluation of Postoptimized Tissue Surrogate–Adjusted Calibration Curves

**Figure 1c** is the plot of all 6 postoptimized tissue surrogate–adjusted calibration curves. For better agreement with the measured WET values, 2 fill-in points were added to the postoptimized tissue surrogate–adjusted calibration curves. One fill-in point was added between the lung-inflated and adipose tissue, and the second was added between the water and the cartilage. The postoptimized tissue surrogate–adjusted calibration curves showed that the high-density bone region had lower SPR values than the preoptimized tissue surrogate–adjusted calibration curves, indicating that the unadjusted stoichiometric calibration may overestimate the SPR for high-density materials. The SPR values for the low-density lung region did not show much difference between the calibration curves of the preoptimized and postoptimized tissue surrogate. Furthermore, the SPR values for the water/tissue region (−100 HU to 100 HU) showed a flatter calibration curve for the postoptimized tissue surrogate than for the preoptimized tissue surrogate. The largest variation (2σ) of the 6 calibration curves was observed for higher-density tissue surrogates for the mandible (2σ = 8.1 HU), cortical bone from ICRU 49 (2σ = 10.2 HU), and cortical bone from ICRU 46 (2σ = 11.1 HU)). The overall average variation (2σ) of HU values for the 6 calibration curves was 5.0 HU, with maximum and minimum HU values of 11.1 HU and 1.1 HU (not counting for the fill-in points), respectively (see **Table 3**). The tabulated postoptimized tissue surrogate–adjusted results of the WET percentage of difference is shown in **Table 2**. The tissues that showed the largest WET percentage of difference were fat (range, −1.5% to −1.4%), stomach (range, 1.5% to 1.6%), and heart (range, 3.0% to 4.4%). The RMSE for the WET percentage of difference after the adjustment for averaged and CT-specific calibration curves was 1.3% and 1.5%, respectively. The overall RMSE for both averaged and CT-specific calibration curves was 1.5%. It was determined that the overall SPR uncertainty after the animal tissue surrogate adjustment was 1.5%.

### Evaluation of Postoptimized Tissue Surrogate–Adjusted Calibration Curve Using Postoptimized Animal Tissues

**Figure 2.**
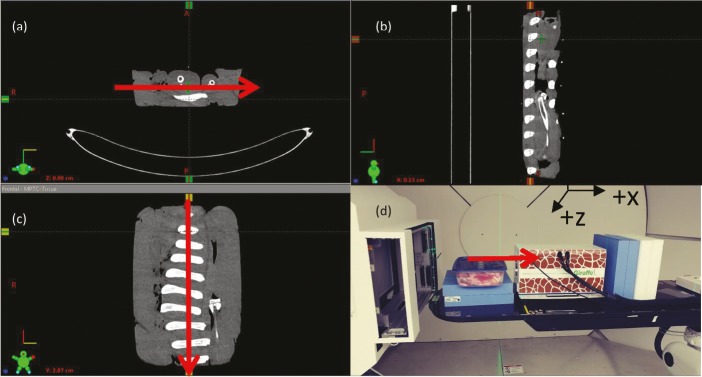
Computed tomography (CT) images of the postoptimized tissue surrogate axis (xy-plane) (a), sagittal (yz-plane) (b), coronal (xz-plane) (c), and measurement setup (d). Red arrows indicate the direction of the proton beam to determine the water-equivalent thickness (WET) value of the postoptimized tissue surrogate. The measured WET value of the tissue surrogate is compared against the treatment planning system WET value to determine the overall uncertainty of the CT calibration curves.

A separate set of animal tissue surrogates was used to evaluate the overall uncertainty of the postoptimized tissue surrogate–adjusted calibration curves. **Figure 2** shows the CT images of the animal tissue surrogate and the measurement setup. **Table 4** shows the tabulated results of the overall uncertainty using the postoptimized tissue surrogate–adjusted calibration curves. The first set of evaluations was performed by applying the averaged CT calibration curves to CT images acquired with each of the individual CT scanners. The second set of evaluations were performed by applying the CT-specific calibration curves to the respective CT images and is shown in **Table 3**. The measured WET values from the separate tissue surrogates were compared against the WET values from the TPS to determine the overall uncertainty of the CT calibration curves. For the averaged CT calibration, the overall SPR uncertainty ranged from −3.7% to 3.4%. The overall RMSE for the average CT calibration was 2.1%. For the CT-specific calibration, the overall SPR uncertainty ranged from −4.3% to 2.4%. The overall uncertainty for each calibration was 2.1%.

## Discussion

### Range Uncertainty

The variability of the stoichiometric calibration curves for 5 CT scanners as well as the average of all 5 individual scanner curves was evaluated. We determined that an averaged CT calibration curve was appropriate for all 5 CT scanners used in this study without increasing the uncertainty in the determination of the proton SPR. Before the tissue surrogate optimization, the overall WET percentage of difference between the TPS values and the measured values ranged from −6.8% to 7.6%, with an RMSE of 4.0%. After the tissue surrogate optimization of all the calibration curves (ie, 5 CT-specific calibration curves and the averaged CT calibration curve), the RMSE for the WET percentage of difference between the TPS values and the measured values was reduced to 1.5%. To evaluate the overall uncertainty of the postoptimized tissue surrogate–adjusted calibration curves, postoptimized tissue surrogates were used. The overall WET percentage of difference (ie, 5 CT-specific calibration curves and the averaged CT calibration curve) ranged from −4.3% to 3.4% with an RMSE of 2.1%. Based on the evaluation of the proton range uncertainty using the postoptimized tissue surrogates, the overall proton range uncertainty of the averaged CT calibration curve after tissue surrogate adjustment in terms of RMSE was 2.1%. Establishing a CT calibration curve using the standard stoichiometric method generated an overall proton range uncertainty of 3% to 4% in this study. To achieve less than 2.5% proton range uncertainty, it has been suggested that studies based on animal tissue surrogates should be performed because of the inherent issue of degeneracy between the HU values and SPRs of human tissues by the stoichiometric method [2].

Within the Department of Radiation Oncology, University of Maryland, there are 5 CT scanners for which a planning CT scan can be performed for proton treatment. Although that is convenient for the patient and the clinical workflow, it poses a challenge when it comes to selecting the appropriate HU-to-SPR calibration curve. To accommodate all 5 CT scanners for proton-planning purposes, there must be multiple HU-to-SPR calibration curves, with the risk of choosing an incorrect calibration curve at the time of planning CT image imported into the TPS. The current study showed that, between the 5 CT scanners, the CT-specific calibration curve showed minimal variation un the HU values (see **Table 2**). Because of the minimal variation in the HU values from the 5 CT scanner, an averaged HU-to-SPR calibration curve can be used to describe the HU-to-SPR relation among the 5 CT scanners, if the CT scanners are properly calibrated and show minimal HU variability.

The fill-in points in the postoptimized tissue surrogate–adjusted calibration curves require some explanation. The 2 fill-in points were required to increase the accuracy of the calibration curve with respect to the measured WET values. The number and placement of fill-in points need to be carefully determined. The fill-in points in this study were selected by evaluating the WET values for multiple tissue samples. The most difficult part of that process is that the fill-in points may improve the WET values for low-density tissues but increase the errors for high-density tissues. For that reason, both the low- and high-density WET values must be determined carefully when selecting such fill-in points. For the set of tissue surrogates, those 2 fill-in points provided the most-optimal calibration curves, which minimized the difference between the TPS-based and measured WET values.

### Comparison of Proton Uncertainty Reported in Literature

Other investigators have studied the overall uncertainty of HU-to-SPR calibration curves. Cheng et al [5] examined the HU-to-SPR calibration curves from 18 CT scanners with 5 RMI 467 phantoms and investigated the effects of dosimetric uncertainties. The HU-to-SPR calibration curves were generated by mapping the scanned HU values from the RMI phantoms and calculating the SPR by Bethe-Block formalism of the tissue substitute inserts in the RMI phantom. The stoichiometric calibration method was not used to generate the calibration curves. To evaluate the dosimetric uncertainty of the calibration curves, the investigators generated 3 calibration curves that represented minimum, maximum, and average HU values for all the curves measured from the scanners. Those curves were compared against the clinically accepted calibration curves at the investigator's institution. Prostate and head-and-neck cases were used to evaluate the dosimetric uncertainties. The investigators reported a dosimetric uncertainty for the prostate plan of 1% for all volumes of interest using the 3 calibration curves (minimum, maximum, and average HU-to-SPR calibration curves) when compared against the clinically accepted calibration curve. For the head-and-neck case, they reported a dosimetric uncertainty of 5% with more than 10% uncertainty for the optic nerves and cochlea. The current study has demonstrated better overall uncertainty (2.1%) using the averaged calibration curve, than was found in the Cheng et al [5] study. Furthermore, Cheng et al [5] evaluated the calibration curves against the clinically accepted calibration curve as their reference. Because they never reported the uncertainty for the reference curve, it is not possible to determine the overall dosimetric uncertainty for the study. Lastly, there was no measurement-based dosimetric uncertainty evaluation of the TPS using the generated calibration curves.

A recent study [13] evaluated dosimetric uncertainty from dual-energy CT (DECT) and single-energy CT (SECT) for proton therapy treatment. A CT phantom was scanned with a SECT at the clinical energy spectrum and scanned again with lower-energy spectrum to generate a complementary DECT image set. Stoichiometric calibration was performed with the SECT and DECT image sets. The HU-to-SPR calibration curves were obtained with the CIRS Model 062M electron density tissue substitute phantom, and the calibration curves were validated with the Catphan module 404 phantom (The Phantom Laboratory, Greenwich, New York) based on vendor-supplied density and elemental composition of the phantoms. No measurements were performed to validate the calibration curves. The investigators reported that the HU-to-SPR calibration for the DECT was more accurate than that of SECT. The maximum-dose calculation error in the SECT plan was 7.8% compared with 1.4% in the DECT plan. The RMSE in the dose calculation was 2.3% and 0.4% for SECT and DECT, respectively, which is slightly worse than our study for the SECT.

Witt et al [14] investigated the difference between the stoichiometric calibration and a calibration curve from beam range measurement with tissue substitutes from phantom model 467 (Gammex, Middleton, Wisconsin). The measurement-based calibration curve was performed by irradiating the tissue substitutes with 284 MeV of carbon ion and measuring the range using a Peakfinder (PTW-Freiburg, Freiburg, Germany). The stoichiometric calibration showed negligible differences up to 150 HU. The slopes of the calibration curves started to diverge from 150 HU to 2500 HU. A favorable agreement was reported up to 1200 HU. Above 1200 HU, the curves proceeded to diverge where the measured curve showed lower SPR values than the stoichiometric curve, which was consistent with our findings. In our study, the posttissue surrogate calibration curves of the high-density bone region had lower SPR values than the stoichiometric calibration curves (see **Figure 1**), in agreement with observations of Witt et al [14], indicating that the stoichiometric calibration method may overestimate the SPR values for high-density materials.

### Significance of Using Animal Tissue Surrogates to Adjust Calibration Curves

In our study, animal tissue surrogates, such as pig parts (pig head, lung, fat, water, stomach, muscle, liver, and femur), significantly improved the uncertainty from 4% to 1.5%. Based on this study, performing stoichiometric calibrations alone was insufficient to reduce the overall uncertainty to less than 3%. The best that could be achieved was typically 3.5% [2]. The reason for such uncertainty is due to several factors. One of the main factors is the variations in the HU of the planning CT from scatter and beam-hardening effects in the patient's anatomy. Minor CT artifacts from high-density materials can cause the TPS to use the wrong SPR values, which could significantly increase the errors in the dose calculation. The errors in the parameterization of the stoichiometric formula to determine the theoretical HUs can add to the overall uncertainty. The stoichiometric calibration requires a fit of measured HU values with known materials to predict the HU of the calculated SPR of the ICRU reference tissues. Uncertainty and error in the fit parameterization will propagate downstream and affect dose calculation as well. Moreover, variation in the patient's anatomic tissue composition from that in the ICRU reference tissues can increase the SPR uncertainty. For instance, femur density will vary from patient to patient, which will affect the SPR value for the same tissue type. In this study, the initial calibration curves were generated with the stoichiometric calibration methods from a tissue substitute from a known phantom. A set of animal tissue surrogates (pig tissue parts) were used not only to validate the calibration curves from the tissue substitutes but also to further optimize the curve for better agreement with the WET measurements. The current study and the Witt et al [14] study show that the stoichiometric calibration method may overestimate the SPR in the high-density region of a curve. One of the best ways to determine the magnitude of the adjustment of the SPR in the high-density region is via measurement. In addition to optimizing the curves, new tissue surrogates were used to evaluate the overall uncertainty of the calibration curves, which is much more comprehensive than simply using another tissue substitute to validate the calibration curves. Because of the inherent errors within the stoichiometric calibration methodology, the current study shows that some type of tissue surrogate evaluation and optimization should be performed to minimize the uncertainty in the calibration curve for the CT scanner in question.

## Conclusion

The current study was performed to evaluate the variability of the stoichiometric HU-to-SPR calibration curves of 5 CT scanners to determine whether an averaged HU-to-SPR calibration curve could be used to account for all 5 CT scanners. Based on the study, the overall average variation in HU values from all the calibration curves before the optimized adjustment of the calibration curves was 8.3 HU. The RMSE of the WET percentage of difference before and after the tissue surrogate optimization for both the averaged and CT-specific calibration curves were 4% and 1.5%, respectively. The overall range of the uncertainty evaluated by the postoptimized tissue surrogate was 2.1% for both averaged and CT-specific calibration curves. The current study shows that an averaged HU-to-SPR calibration curve can be used to account for variability among CT scanners, if CT scanners are properly calibrated and show minimal HU variability from one CT scanner to another. Lastly, to achieve overall proton range uncertainty of less than 2%, we have demonstrated that an animal tissue surrogate–based optimization of the HU-to-SPR calibration curve should be performed.
